# Using an influenza surveillance system to estimate the number of SARS-CoV-2 infections in Beijing, China, weeks 2 to 6 2023

**DOI:** 10.2807/1560-7917.ES.2023.28.11.2300128

**Published:** 2023-03-16

**Authors:** Li Zhang, Yi Zhang, Wei Duan, Shuangsheng Wu, Ying Sun, Chunna Ma, Quanyi Wang, Daitao Zhang, Peng Yang

**Affiliations:** 1 Beijing Center for Disease Prevention and Control, Dongcheng District, Beijing, China; 2 General Administration of Customs (Beijing) International Travel Health Care Center, Dongcheng District, Beijing, China

**Keywords:** COVID-19, SARS-CoV-2, Multiplier model, Surveillance

## Abstract

With COVID-19 public health control measures downgraded in China in January 2023, reported COVID-19 case numbers may underestimate the true numbers after the SARS-CoV-2 Omicron wave. Using a multiplier model based on our influenza surveillance system, we estimated that the overall incidence of SARS-CoV-2 infections was 392/100,000 population in Beijing during the 5 weeks following policy adjustment. No notable change occurred after the Spring Festival in early February. The multiplier model provides an opportunity for assessing the actual COVID-19 situation.

From early December 2022, the coronavirus disease (COVID-19) epidemic greatly impacted major cities in China, including Beijing [1], as a result of the predominant and highly transmissible severe acute respiratory syndrome coronavirus 2 (SARS-CoV-2) Omicron (Phylogenetic Assignment of Named Global Outbreak (Pango) lineage designation: B.1.1.529) subvariant BF.7 [2-4]. Following the adjustment of China’s ‘zero-COVID’ policy, which included the termination of universal nucleic acid testing, the accurate number of SARS-CoV-2 infections was challenging to determine. Here, we used the influenza-like illness (ILI) surveillance platform combined with laboratories of ILI virologic surveillance to understand the prevalence of SARS-CoV-2. We estimated the actual number of infected individuals in Beijing from the first week after the downgraded management of COVID-19 (week 2) to 2 weeks after the Spring Festival holiday (week 6) in 2023 using a multiplier model.

## COVID-19 management in Beijing

Based on high COVID-19 vaccine coverage, accumulated experience in prevention and treatment, and the shift of SARS-CoV-2 variant characteristics to high transmissibility and less virulence, the strategy of COVID-19 management in China switched from a ‘zero-COVID’ policy (in place for about 3 years) to a more relaxed scenario after the SARS-CoV-2 Omicron wave. From 8 January 2023 (week 2), China downgraded the management of COVID-19 in accordance with the country's law on prevention and treatment of infectious disease [5]. With the policy adjustment, universal nucleic acid testing of SARS-CoV-2 for the general population was terminated. As a result, the reported COVID-19 case data after this time may underestimate the true number of infections. To understand the size of SARS-CoV-2-infected population to be able to allocate healthcare resources, an estimation of the number of infections is important. In addition, increased gatherings and the mass mobility on account of the week-long Spring Festival holiday (week 4) required us to closely monitor the trend of COVID-19 during this period.

## Influenza surveillance system adaptation

We estimated COVID-19 case numbers based on the influenza surveillance system in Beijing. This system covers all levels of local hospitals, and has been in operation since 2007 [6]. The stable and qualified database output from the surveillance system made it an ideal tool for other respiratory diseases surveillance [7,8]. 

To monitor the COVID-19 epidemic using our influenza surveillance system, we integrated SARS-CoV-2 testing into the existing ILI virological surveillance. ILI was defined as fever ≥ 38 °C and cough or sore throat. The collected pharyngeal swab specimens of ILIs transported to collaborating laboratories were tested for both influenza viruses and SARS-CoV-2 by real-time reverse transcription PCR (rRT-PCR).

To estimate the true number of SARS-CoV-2 infections (both asymptomatic and symptomatic infections) from week 2 to week 6 2023 in Beijing, we used a revised model based on the multiplier model method developed by Reed et al. [9]. The revised model method has been previously used to estimate the disease burden of pandemic A(H1N1) pdm09 virus and seasonal influenza virus infections in China [10,11].

## Estimation of true SARS-CoV-2 infections

Multiplier models use probabilistic multipliers to adjust for sources of under-ascertainment to better estimate the true burden of a pathogen. In this study, five parameters were included to adjust COVID-19 cases counts in the model (Figure and Table 1): (A) the proportion of symptomatic patients among SARS-CoV-2 infections, (B) the proportion of ILI among symptomatic SARS-CoV-2 infections, (C) the consultation rate among ILI patients with SARS-CoV-2 infection, (D) the success rate for sampling pharyngeal swab specimens and (E) the test sensitivity of detection using PCR. Parameters were identified by prior published studies and the recent surveys on SARS-CoV-2 infections (Table 1). Values for parameters A–C were obtained through a community sampling survey on SARS-CoV-2 infections conducted by the Beijing Center for Disease Prevention and Control during 8 and 9 January 2023, which sampled about 17,000 individuals from all districts of Beijing. SARS-CoV-2 infection in the survey was defined as testing positive for SARS-CoV-2 nucleic acid or antigen. Multipliers were calculated as the inverse of the product of parameters A–E.

**Figure:**
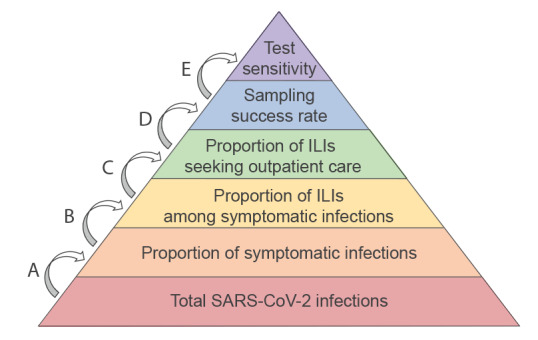
Model parameters for estimating the true number of SARS-CoV-2 infections, Beijing, China, weeks 2 to 6 2023

ILI: influenza-like illness; SARS-CoV-2: severe acute respiratory syndrome coronavirus 2.

Both asymptomatic and symptomatic SARS-CoV-2 infections are considered.

**Table 1:** Parameter values and sources of data included in the multiplier model for estimating the number of SARS-CoV-2 infections, Beijing, China, weeks 2 to 6 2023

Code	Parameter	Value	Data source
A	Proportion of symptomatic patients among SARS-CoV-2 infections	87.6–94.2%	Community survey of SARS-CoV-2 infections conducted by Beijing CDC
B	Proportion of ILI among symptomatic SARS-CoV-2 infections^a^	50.6–63.3%	Community survey of SARS-CoV-2 infections conducted by Beijing CDC
C	Consultation rate among ILIs with SARS-CoV-2 infection^a^	8.4%	Community survey of SARS-CoV-2 infections conducted by Beijing CDC
D	Success rate for sampling pharyngeal swab specimens	80–90%	Published study [10]
E	Test sensitivity of detection of PCR	95–100%	Published study [10]

CDC: Center for Disease Prevention and Control; ILI: influenza-like illness; SARS-CoV-2: severe acute respiratory syndrome coronavirus 2.

The values indicate parameters used in the model.

^a^The definition of ILI is patients presenting with fever ≥ 38 °C with cough or sore throat.

The true number of SARS-CoV-2 infections was calculated by multiplying the baseline number of patients with SARS-CoV-2 infection by multipliers. The baseline number was equal to the product of the reported weekly number of ILI consultations and the weekly positive rate of SARS-CoV-2. Data of ILI consultations were collected from the ILI surveillance system, and proportions of consultations by age group were calculated. The weekly positive rate of SARS-CoV-2 was obtained through virological surveillance. The overall true number of SARS-CoV-2 infections and the true number by age group (0–4 years, 5–14 years, 15–24 years, 25–59 years, and ≥ 60 years) were estimated. Using a Monte Carlo approach, each parameter included in the model was assigned a uniform probability distribution, from which the model randomly sampled 1,000 iterations. The median and 95% uncertainty intervals (UI; percentiles 2.5 to 97.5) were obtained from 1,000 simulations. The incidence of infections per 100,000 individuals was estimated by dividing the number of these outcomes by the size of the population.

The overall estimated number of SARS-CoV-2 infections was 85,766 (95% UI: 74,514–100,477) from week 2 to week 6, 2023 in Beijing (Table 2). The infection number was highest among adults aged 25–59 years (n = 32,626; 95% UI: 28,346–38,222), and was the lowest among younger adults aged 15–24 years (n = 6,518; 95% UI: 5,663–7,636). Results of weekly estimated number of SARS-CoV-2 infections showed that the new infections per week dropped steadily during week 2 to week 4, from 49,003 (95% UI: 43,741–56,176) in week 2 to 5,372 (95% UI: 4,729–6,119) in week 4. The estimated number increased slightly in week 5 (n = 5,836; 95% UI: 5,084–6,744), and remained at a similar level in week 6 (n = 5,701; 95% UI: 4,927–6,614). A similar trend was found in all age groups (Table 2).

**Table 2:** Estimated numbers and incidence rates of SARS-CoV-2 infections by age groups and by week, Beijing, China, week 2 to week 6 2023 (n = 85,766)

Age group (years)	Estimated number of SARS-CoV-2 infections	95% UI	Estimated incidence per 100,000 population^a^	95% UI
Total				
0–4	12,530	10,886–14,680	1,233	1,072–1,444
5–14	11,407	9,910–13,363	724	629–848
15–24	6,518	5,663–7,636	329	286–385
25–59	32,626	28,346–38,222	251	218–294
≥ 60	22,685	19,709–26,576	528	459–618
Overall	85,766	74,514–100,477	392	340–459
Week 2				
0–4	4,846	4,326–5,556	477	426–547
5–14	4,753	4,243–5,449	302	269–346
15–24	3,381	3,018–3,876	170	152–195
25–59	19,803	17,676–22,701	152	136–174
≥ 60	16,220	14,478–18,594	377	337–433
Overall	49,003	43,741–56,176	224	200–257
Week 3				
0–4	2,511	2,215–2,849	247	218–280
5–14	2,318	2,045–2,630	147	130–167
15–24	1,263	1,114–1,433	64	56–72
25–59	7,644	6,742–8,672	59	52–67
≥ 60	6,097	5,377–6,917	142	125–161
Overall	19,833	17,493–22,501	91	80–103
Week 4				
0–4	1,079	950–1,228	106	93–121
5–14	735	647–838	47	41–53
15–24	335	294–381	17	15–19
25–59	1,889	1,663–2,152	15	13–17
≥ 60	1,334	1,175–1,520	31	27–35
Overall	5,372	4,729–6,119	25	22–28
Week 5				
0–4	1,086	946–1,255	107	93–123
5–14	999	870–1,155	63	55–73
15–24	554	483–640	28	24–32
25–59	2,107	1,836–2,435	16	14–19
≥ 60	1,090	949–1,259	25	22–29
Overall	5,836	5,084–6,744	27	23–31
Week 6				
0–4	1,068	923–1,238	105	91–122
5–14	1,081	935–1,254	69	59–80
15–24	570	492–661	29	25–33
25–59	2,088	1,805–2,424	16	14–19
≥ 60	894	772–1,037	21	18–24
Overall	5,701	4,927–6,614	26	23–30

SARS-CoV-2: severe acute respiratory syndrome coronavirus 2; UI: uncertainty interval.

Both asymptomatic and symptomatic SARS-CoV-2 infections are considered. The Spring Festival holiday of 2023 was in week 4.

^a ^The population of Beijing used for estimation is from the seventh national population census of China, which is 21.89 million.

According to the population size of Beijing [12], the overall incidence was 392 per 100,000 people (95% UI: 340–459) from week 2 to week 6, 2023 in Beijing, with the highest incidence rate among children aged 0–4 years (1,233/100,000; 95% UI: 1,072–1,444) and the lowest incidence rate among adults aged 25–59 years (251/100,000; 95% UI: 218–294) (Table 2).

## Discussion

We estimated that around 86,000 SARS-CoV-2 infections might have occurred in Beijing from week 2 to week 6 2023, with the incidence rate of approximately 400 per 100,000 population. This result suggested that after the downgraded management, the COVID-19 epidemic situation in Beijing entered a low level, and did not significantly rebound even after the Spring Festival holiday in early February despite increased gatherings and mass population mobility. Through analysis by age group, we found that the incidence rates of children (aged 0–4 years) and older adults (aged ≥ 60 years) were higher relative to those of adults aged 25–59 years.

We found that the incidence among 0–4-year-old children was higher compared to other age groups during the study period. We hypothesise that the infections among younger children might have occurred later than other age groups because parents imparted specific precautions to their children to prevent them from being exposed/infected during the Omicron wave. Firstly, children's outdoor activity might be reduced during the period from December 2022 to early January 2023, and they were mainly infected by family members [13,14]. Therefore, the infection risk of children was lower than that of adults during that period, and the infection time could be relatively lagged. Secondly, with the social activity restrictions lifted and the decline of COVID-19 epidemic intensity, children's outdoor activity increased from early January 2023, which may lead to an increased risk of infection through social contact.

The multiplier model has been applied to estimate the incidence of COVID-19 effectively in previous studies [16,17]. Activity of SARS-CoV-2 in our sentinel surveillance was consistent with the trend of national data [1], and was in line with the estimation of the transmissibility of SARS-CoV-2 Omicron subvariant BF.7 in Beijing [15]. This consistency and the robust performance of the surveillance system previously demonstrated in estimating true infections of pandemic A(H1N1) pdm09 and seasonal influenza enabled us to conduct similar estimation for COVID-19. It is unfortunate that the infection number of SARS-CoV-2 before the measures were relaxed could not be estimated using the multiplier model approach, since the weekly virological surveillance for COVID-19 started to be conducted from week 2 of 2023, after policy adjustment. The accuracy of our estimated true number of SARS-CoV-2 infections should be further verified by other methods, such as telephone/online surveys or serological research. In addition, parameters used in the model should be refined based on the additional data from surveys in the future, since data for parameter estimates were collected in limited periods in this study.

## Conclusions

To the best of our knowledge, this is the first work using the multiplier model method to estimate the true number of SARS-CoV-2 infections in China by integrating COVID-19 into the existing influenza surveillance system. This method met our need of daily monitoring and enabled us to assess the epidemic level of COVID-19. Continued optimisation of this method is needed to build a long-term COVID-19 surveillance for monitoring potential future waves of epidemic. Our study provided deep insights into the role of a multiplier model and existing surveillance system in assessing actual situation of COVID-19 or other emerging respiratory pathogens for other regions in China as well as other countries.
